# Epidemiology of *Haemophilus ducreyi* Infections

**DOI:** 10.3201/eid2201.150425

**Published:** 2016-01

**Authors:** Camila González-Beiras, Michael Marks, Cheng Y. Chen, Sally Roberts, Oriol Mitjà

**Affiliations:** Nova University of Lisbon, Lisbon, Portugal (C. González-Beiras);; Barcelona Institute for Global Health, Barcelona, Spain (C. González-Beiras, O. Mitjà);; London School of Hygiene and Tropical Medicine, London, UK (M. Marks);; Hospital for Tropical Diseases, London (M. Marks);; Centers for Disease Control and Prevention, Atlanta, Georgia, USA (C.Y. Chen);; Auckland District Health Board, Auckland, New Zealand (S. Roberts);; Lihir Medical Centre, Lihir Island, Papua New Guinea (O. Mitjà)

**Keywords:** Haemophilus ducreyi, bacteria, epidemiology, chancroid, genital ulcer disease, genital ulcers, skin ulcers, nongenital cutaneous infections, sexually transmitted infections

## Abstract

The global epidemiology of *Haemophilus ducreyi* infections is poorly documented because of difficulties in confirming microbiological diagnoses. We evaluated published data on the proportion of genital and nongenital skin ulcers caused by *H. ducreyi* before and after introduction of syndromic management for genital ulcer disease (GUD). Before 2000, the proportion of GUD caused by *H. ducreyi* ranged from 0.0% to 69.0% (35 studies in 25 countries). After 2000, the proportion ranged from 0.0% to 15.0% (14 studies in 13 countries). In contrast, *H. ducreyi* has been recently identified as a causative agent of skin ulcers in children in the tropical regions; proportions ranged from 9.0% to 60.0% (6 studies in 4 countries). We conclude that, although there has been a sustained reduction in the proportion of GUD caused by *H. ducreyi*, this bacterium is increasingly recognized as a major cause of nongenital cutaneous ulcers.

## Abstract 2

Infections are at their lowest level worldwide, but nongenital cutaneous infections have increased.

*Haemophilus ducreyi*, a fastidious gram-negative bacterium, is the causative agent of chancroid, a genital ulcer disease (GUD). The organism is usually spread during sexual intercourse through microabrasions, and the disease usually manifests as multiple painful superficial ulcers associated with inguinal lymphadenitis (*1*). As a result of the painful nature of the lesions, patients usually seek immediate treatment, and asymptomatic carriage is therefore uncommon (*2*). In addition to causing GUD, *H. ducreyi* has been found in several recent studies to be a major cause of chronic skin ulceration in children from developing countries (*3*–*6*).

The global epidemiology of chancroid is poorly documented, and it is not included in World Health Organization estimates of the global incidence of curable sexually transmitted infections (STIs). There are some key challenges in interpreting data on the epidemiology of *H. ducreyi* as a causative agent of GUD. First, genital herpes cases are easily misdiagnosed as chancroid on clinical examination. Thus, reports based only on clinical diagnosis can be erroneous. Second, laboratory culture is technically difficult, and the highly sensitive and specific nucleic acid amplification tests, such as PCR, are rarely available outside national reference laboratories or specialized STI research settings, which makes it difficult to confirm clinical diagnoses.

Determination of the true global incidence of chancroid is made more difficult by widespread adoption of syndromic management for bacterial GUD (i.e., treatment with antimicrobial drugs effective against syphilis and chancroid) without microbiological confirmation in many countries. Therefore, countries often report only the total number of GUD cases. In addition, identification of GUD etiology is rarely conducted in resource-poor countries to validate syndromic management for which chancroid could also be common.

Earlier studies of tropical skin ulcers did not generally test for *H. ducreyi,* with the exception of a small number of case reports (*7**–**11*). There are major limitations in describing the prevalence of causative agents in tropical skin lesions that typically occur in children in rural areas where there is no access to laboratory facilities. Pathogens such as *Fusobacterium fusiforme, Staphylococcus aureus*, and *Streptococcus pyogenes* have been reported from Gram staining of exudative material collected from tropical ulcers (*12*). However, cultures or PCR testing for definitive identification of fastidious pathogens involved has not been traditionally conducted. The purpose of this study was to improve our understanding of the epidemiology of *H. ducreyi* infection through a systematic review of published data on the proportion of genital and skin ulcers caused by this bacterium.

## Methods

### Search Strategy and Selection Criteria

A systematic review was conducted to identify all relevant studies that examined the etiology of GUD and nongenital skin ulcers involving *H. ducreyi*. We searched the National Library of Medicine through PubMed for “*H. ducreyi*,” “chancroid,” “genital ulcer,” OR “skin ulceration” AND “proportion” OR “prevalence.” The search was limited to studies published during January 1, 1980–December 31, 2014. In addition, we searched references of identified articles and other databases for other articles, and we reviewed abstracts, titles, and selected studies potentially containing information on chancroid epidemiology. We contacted researchers who were working with *H. ducreyi* to identify unpublished literature for inclusion. No language restrictions were set for searches.

**Figure 1:**
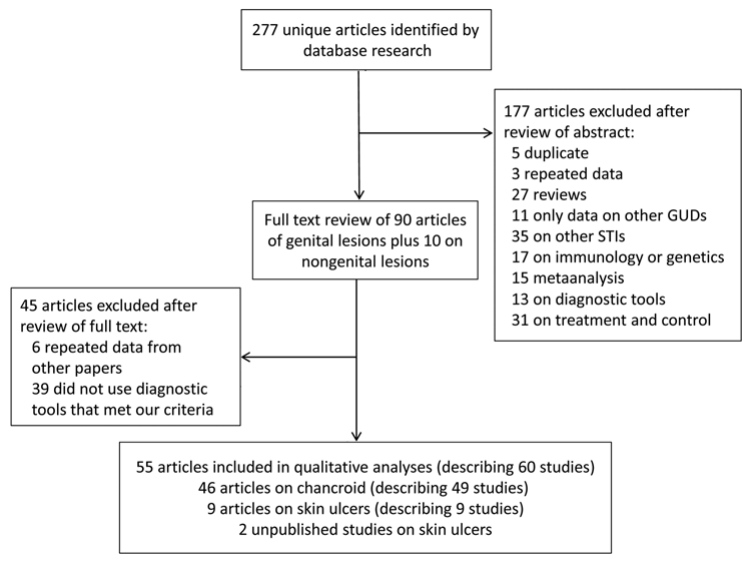
Procedure for selecting eligible references on the epidemiology of *Haemophilus ducreyi* as a causative agent of genital ulcers. GUDs, genital ulcer disease; STI, sexually transmitted infections.

**Figure 2:**
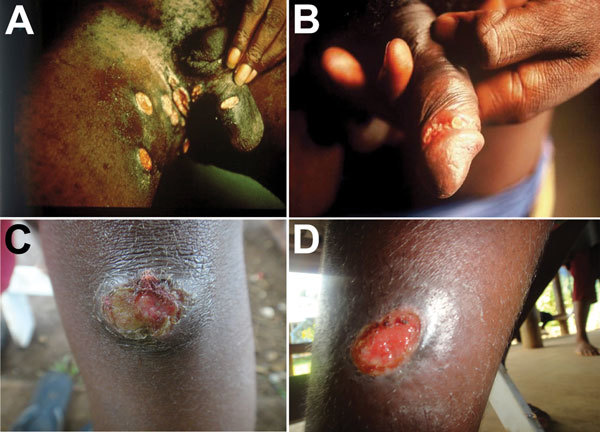
Ulcers caused by infection with *Haemophilus ducreyi*. A, B) Genital ulcers in adult patients from Ghana (provided by David Mabey). C, D) Skin ulcers in children from Papua New Guinea (provided by Oriol Mitjà).

The decision tree for inclusion or exclusion of articles is shown in Figure 1. We included studies if the proportion of etiologic agents in genital ulcers and nongenital skin ulcers, including *H. ducreyi*, was confirmed by laboratory techniques. Clinical diagnosis of chancroid is often based on the appearance of the ulcer, which is characteristically painful, purulent, and deep with ragged, undermined edges (Figure 2). However, because the appearance of these ulcers is similar to ulcers caused by other bacteria, clinical diagnosis can be nonspecific or insensitive and often requires laboratory confirmation (*1*). In addition, microscopy identification of typical morphologic features and serologic detection lack sensitivity and specificity (*13*,*14*). Thus, we only considered the following diagnostic methods as providing acceptable evidence of *H. ducreyi* infection: 1) isolation and identification by culture; or 2) PCR/real-time PCR.

### Data Extraction and Synthesis

For all qualifying studies, extracted data included study country, year of study, diagnostic test used for confirmation, total number of *H. ducreyi*–positive cases, and sample size. Descriptive analyses of extracted data were conducted, and the number of *H. ducreyi*–confirmed cases was divided by the total number of cases to calculate the proportion of cases caused by *H. ducreyi.* Studies qualifying for data extraction were grouped into 2 categories: studies conducted before 2000 and studies after 2000. This date separates studies before and after widespread implementation of syndromic management of GUD. Study sites were also plotted by geographic region. No quantitative metaanalysis was undertaken.

## Results

We identified 277 records in which we found 46 articles describing 49 studies on GUD that met our inclusion criteria (Tables 1, 2; Technical Appendix). All identified studies were based on cohorts of patients attending STI clinics, including 3 studies that enrolled only commercial sex workers. The age group for all cases was adults >18 years of age, except for 3 studies in Zambia, South Africa, and China, which included patients >16 years of age, and 1 study in Madagascar, which included patients >14 years of age. A total of 9 published studies and 2 unpublished reports that described nongenital skin ulcers caused by *H. ducreyi* were also included in our systematic review.

**Table 1:** Characteristics of 35 studies of genital ulcers caused by *Haemophilus ducreyi*, 1980–1999*

Area, reference†	Country	Year of study	Diagnostic method	No. patients with GUD	No. cases *H. ducreyi* infection	% (95% CI)
Africa						
Paz-Bailey et al. (*16*)	Botswana	1993	Culture	108	27	25.0 (17.7–33.9)
Steen (*17*)	Côte d’Ivoire	1996	PCR	NA	NA	47
Mabey et al. (*18*)	Gambia	1987	Culture	104	54	51.9 (42.4–61.2)
Hawkes et al. (*19*)	Gambia	1995	M-PCR	18	8	44.4 (24.5–66.2)
Nsanze et al. (*20*)	Kenya	1980	Culture	97	60	61.8 (51.9–70.9)
Kaul et al. (*21*)	Kenya	1997	Culture	189	54	28.5 (22.6–35.3)
Morse et al. (*22*)	Lesotho	1994	M-PCR	105	55	53.3 (43.8–62.6)
Harms et al. (*23*)	Madagascar	1992	Culture	12	61	19.6 (11.6–31.3)
Behets et al. (*24*)	Madagascar	1997	M-PCR	196	64	32.6 (26.4–39.5)
Behets et al. (*25*)	Malawi	1995	M-PCR	778	204	26.2 (23.2–29.4)
Hoyo et al. (*26*)	Malawi	1999	M-PCR	137	41	29.0 (22.8–38.0)
Bogaerts et al. (*27*)	Rwanda	1992	Culture	395	115	29.1 (24.8–33.7)
Totten et al. (*28*)	Senegal	1992	PCR	39	22	56.4 (40.9–70.7)
Crewe-Brown et al. (*29*)	South Africa	1981	Culture	100	45	45 (35.5–54.7)
Dangor et al. (*30*)	South Africa	1989	Culture	240	164	68.3 (62.2–73.8)
Cheng et al. (*31*)	South Africa	1994	M-PCR	538	171	31.7 (27.9–35.8)
Lai et al. (*32*)	South Africa	1994	M-PCR	160	232	68.9 (62.7–74.5)
	South Africa	1998	M-PCR	94	186	50.5 (43.4–57.6)
Meheus et al. (*33*)	Swaziland	1979	Culture	155	68	43.8 (36.3–51.7)
Ahmed et al. (*34*)	Tanzania	1999	PCR	102	12	11.7 (6.8–19.4)
Le Bacq et al. (*35*)	Zimbabwe	1991	Culture	90	22	24.4 (16.7–34.2)
Asia						
Wang et al. (*36*)	China	1999	M-PCR	96	0	0.0 (0.0–3.8)
Risbud et al. (*37*)	India	1994	M-PCR	302	84	27.8 (23.0–33.1)
Rajan et al. (*38*)	Singapore	1983	Culture	670	56	8·3 (6·4–10·7)
Beyrer et al. (*15*)	Thailand	1996	M-PCR	38	0	0.0 (0.0–9.1)
North America						
Dillon et al. (*39*)	United States	1990	Culture	82	27	32.9 (23.7–43.6)
Mertz et al. (*40*)	United States	1995	M-PCR	143	56	39.1 (231.5–47.3)
Mertz et al. (*41*)	United States	1996	M-PCR	516	16	3.1 (1.9–4.9)
South America						
Sanchez et al. (*42*)	Peru	1995	M-PCR	61	3	4.9 (1.6–13.4)
Caribbean						
Sanchez et al. (*42*)	Dominican Republic	1996	M-PCR	81	21	25.9 (17.6–36.4)
Behets et al. (*43*)	Jamaica	1996	M-PCR	304	72	23·6 (19.2–28.7)
Bauwens et al. (*44*)	Bahamas	1992	PCR	47	7	14·8 (7.4–27.6)
Middle East						
Madani et al. (*45*)	Saudi Arabia	1999	Culture	3,679	78	2.1 (1.7–2.5)
Europe						
Kyriakis et al. (*46*)	Greece	1996	Culture	695	32	4.6 (3.2–6.4)
Bruisten et al. (*47*)	The Netherlands	1996	M-PCR	368	3	0.8 (0.2–2.3)

*GUD, genital ulcer disease; NA, not available; M-PCR, multiplex PCR. †References *41–47* provided in the online Technical Appendix (http://wwwnc.cdc.gov/EID/article/22/1/15-0425-Techapp1.pdf).

**Table 2:** Characteristics of 14 studies of genital ulcers caused by *Haemophilus ducreyi*, 2000–2014*

Area, reference†	Country	Year of study	Diagnostic method	No. patients with GUD	No. cases *H. ducreyi* infection	% (95% CI)
Africa						
Paz-Bailey et al. (*16*)	Botswana	2002	PCR	137	1	0.7 (0.1–4.0)
Mehta et al. (*48*)	Kenya	2007	M-PCR	59	0	0.0 (0.0–6.1)
Phiri et al. (*49*)	Malawi	2006	M-PCR	398	60	15.0 (11.8–18.9)
Zimba et al. (*50*)	Mozambique	2005	PCR	79	3	3.8 (1.3–10.9)
Tobias et al. (*51*)	Namibia	2007	PCR	199	0	0.0 (0.0–1.8)
O’Farrell et al. (*52*)	South Africa	2004	M-PCR	162	2	1.2 (0.3–4.6)
Lewis et al. (*53*)	South Africa	2006	M-PCR	613	10	1.6 (0.9–2.9)
Nilsen et al. (*54*)	Tanzania	2001	PCR	232	12	5.1 (2.9–8.8)
Suntoke et al. (*55*)	Uganda	2006	M-PCR	100	2	2.0 (0.5–7.0)
Makasa et al. (*56*)	Zambia	2010	PCR	200	0	0 (0.0–1.8)
South America						
Gomes Naveca et al. (*57*)	Brazil	2009	PCR	434	0	0 (0.0–0.8)
Middle East						
Maan et al. (*58*)	Pakistan	2009	Culture	521	20	3.8 (2.5–5.8)
Europe						
Hope-Rapp et al. (*59*)	France	2005	Culture	278	8	2.8 (1.4–5.5)
Oceania						
Mackay et al. (*60*)	Australia	2002	M-PCR	64	0	0.0 (0.0–5.6)

*GUD, genital ulcer disease; M-PCR, multiplex PCR. †References *48–60* provided in the online Technical Appendix (http://wwwnc.cdc.gov/EID/article/22/1/15-0425-Techapp1.pdf).

Laboratory confirmation of chancroid by PCR or culture was reported in 33 (67%) and 16 (32%) of the 49 studies, respectively. Of 16 studies that used culture, 7 (43%) used Mueller-Hinton agar with a nutritional supplement (e.g., IsoVitalex; Becton Dickinson, Franklin Lakes, NJ, USA), 1% used hemoglobin, and 5 (31%) used chocolate agar–based media; the remaining studies used other culture media. Five (31%) of 16 studies incubated agar plates at low temperatures (33°C–35°C), and 2 (12%) incubated plates at 36°C. Remaining articles did not specify incubating temperature.

Different PCR primer targets were used to amplify DNA sequences, including the 16S rRNA gene, the *groEL* gene, and the hemolysin gene. In addition to herpes simplex virus (HSV) PCR, 23 studies used a multiplex PCR that could simultaneously detect the 3 major causes of GUD (*H. ducreyi*, *Treponema pallidum*, and HSV types 1 and 2) (*15*). Studies encompassed 33 countries: 17 in Africa, 4 in Southeast Asia, 3 in Europe, 2 in the Middle East, 3 in South America, and 2 in the Caribbean, 1 in the United States, and 1 in Australia.

### Incidence of Chancroid

**Figure 3:**
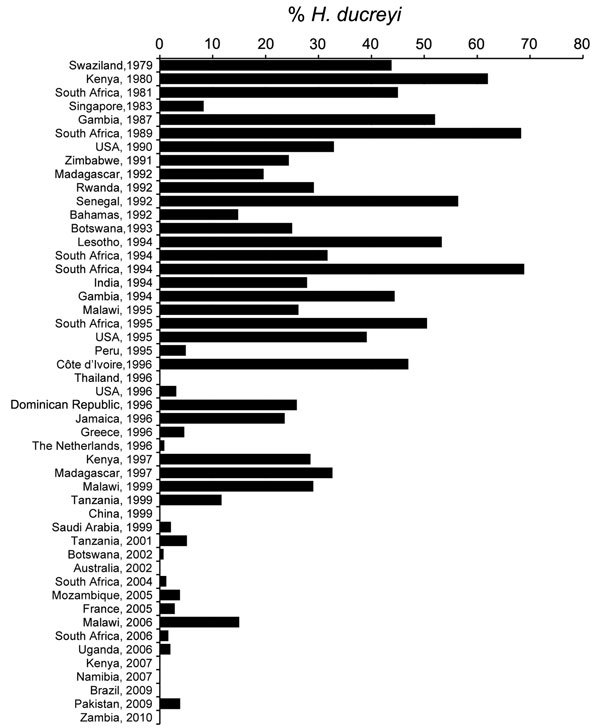
Trend of proportion of genital ulcers caused by infections with *Haemophilus ducreyi,* 1979–2010.

Of 49 studies on chancroid analyzed, 35 were published during 1980–1999 (Table 1) and 14 during 2000–2014 (Table 2). In general, data showed a clear decrease in the proportion of chancroid during 1980–2014 in all areas analyzed (Figure 3).

During 1980–1999, the proportion of genital ulcers caused by *H. ducreyi* in these studies ranged from 0.0% in Thailand and China to 68.9% in South Africa (Table 1). Eleven (31.4%) studies reported high proportions (>40%) of cases of infection with *H. ducreyi*. All of these studies were conducted in countries in Africa (Côte d’Ivoire, Gambia, Kenya, Lesotho, Senegal, South Africa, and Swaziland). Slightly lower proportions (20%–40% of cases) were observed in 15 (42%) studies: 10 in countries in Africa, 2 in the United States during localized outbreaks, 1 in Jamaica, 1 in the Dominican Republic, and 1 in India.

Only a few countries reported low proportions (<10%) of genital ulcers infected with *H. ducreyi*, including Singapore (8.3%), Peru (5%), Greece (4.6%), the Netherlands (0.9%), United States (3.1%), and Saudi Arabia (2.1%). The study in Saudi Arabia was conducted during 1995–1999; a total of 27,490 patients were examined for STIs. Chancroid was diagnosed by culture and was reported as the least common STI during this survey. The only studies that reported no cases of chancroid were conducted in Thailand in 1996 and China in 1999; both studies used multiplex PCR for detection of GUD cases.

During 2000–2014, the proportion of *H. ducreyi* infections was low (<10%) in all studies analyzed, except for 1 study in Malawi (15%) (Table 2). Studies in 5 countries (Kenya, Namibia, Zambia, Brazil, and Australia) did not report any cases of infection with *H. ducreyi*. Other studies reporting proportions of infections <10% were conducted in Botswana, Mozambique, South Africa, Uganda, Pakistan, and France. No reports were found for studies in North America, Southeast Asia, or the Caribbean.

### Nongenital Skin Infections with *H. ducreyi*

During 1988–2010, several case reports described 4 children and 4 adults with nonsexually transmitted infections with *H. ducreyi* that manifested as lower leg lesions but no genital lesions. The reported case-patients were travelers who had been to Fiji (*7*), Samoa (*8*), Vanuatu (*9*), or Papua New Guinea (*10*) (Table 3). Outside the south Pacific region, a 5-year-old refugee from Sudan who had lower leg ulceration was also given a diagnosis of infection with *H. ducreyi* (*11*).

**Table 3:** Characteristics of 11 studies on skin ulcers caused by *Haemophilus ducreyi*, 1988–2014*

Reference	Country	Year of study	Diagnostic method	No. patients with skin ulcers	No cases *H. ducreyi* infection	% (95% CI)
Marckmann et al. (*7*)	Fiji Islands	1988	Culture	1 man	1	NA
Ussher et al. (*8*)	Samoa	2005	PCR	3 girls <10 y of age	3	NA
McBride et al. (*9*)	Vanuatu	2007	PCR	1 woman	1	NA
Peel et al. (*10*)	Vanuatu and Papua New Guinea	2010	PCR	2 men	2	NA
Humphrey et al. (*11*)	Sudan	2007	PCR	1 boy	1	NA
Mitjà et al. (*3*)	Papua New Guinea	2013	PCR	90	54	60.0 (49.6–69.5)
Mitjà et al. (*6*)	Papua New Guinea	2014	PCR	114	60	60.1 (54.3–65.5)
Marks et al. (*4*)	Solomon Islands	2013	PCR	41	13	31.7 (19.5–46.9)
Chen et al.†	Vanuatu	2013	PCR	176	68	38.6 (31.7–46.0)
Chen et al.†	Ghana	2013	PCR	179	49	27.3 (21.3–34.3)
Ghinai et al. (*5*)	Ghana	2014	PCR	90	8	8.8 (4.5–16.5)

*NA, not applicable. †Pers. comm.

A cohort study conducted in Papua New Guinea in 2014 showed evidence that *H. ducreyi* is a major cause of chronic skin ulceration; *H. ducreyi* DNA was identified by PCR in 60.0% of skin lesions in children (*3*). Similar studies in other areas reported laboratory-confirmed skin ulcers in children caused by *H. ducreyi* in Papua New Guinea (*6*), Solomon Islands (*4*), Vanuatu (C.Y. Chen, pers. comm.), and Ghana (*5*) (Table 3).

## Discussion

Our review confirmed 2 major findings. First, reduction in the proportion of genital ulcers caused by *H. ducreyi* has been sustained for the past decade and a half. Second, there is increasing evidence that *H. ducreyi* is a common and newly recognized causative agent of chronic skin ulceration in children from developing countries.

In the 1990s, the global prevalence of chancroid was estimated to be 7 million (*17*). Chancroid was one of the most prevalent GUDs, particularly in resource-poor countries in Africa, Asia, Latin America, and the Caribbean (*1*; reference *51* in Technical Appendix). Recommendations to introduce syndromic management for treatment of GUD caused by bacteria were published by the World Health Organization in 1991 and fully implemented by 2000 (reference *61* in Technical Appendix). Since that time, global incidence of GUDs, particularly chancroid, has decreased substantially, and genital herpes viruses (HSV-1 and HSV-2) have become the predominant cause of GUD (reference *53* in Technical Appendix). Currently in Europe and the United States, chancroid is restricted to rare sporadic cases. Transmission of *H. ducreyi* remains ongoing in only a few countries that have limited access to health services (*2**,**6*).

Our data show marked decreases in the proportion of GUD caused by *H. ducreyi* in several countries. Spinola et al. reported similar conclusions obtained from 25 PCR-based studies (reference *62* in online Technical Appendix). For example, in Botswana (*16*), Kenya, (*20*), and South Africa (*29*), the proportion of GUD caused by *H. ducreyi* decreased from 25%–69% to negligible (0.0%–1.2%) levels (*16*; references *48*,*52* in Technical Appendix). Studies in Zambia (reference *56* in Technical Appendix), Namibia (reference *51* in Technical Appendix), and China (*36*) did not report any cases of chancroid during 2000–2009. A study in Thailand reported elimination of chancroid by introduction of a condom use program in the 1990s (reference *63* in Technical Appendix). Similar decreases have been reported from Cambodia and Sri Lanka, with rapid elimination of chancroid and congenital syphilis in most settings (reference *63* in Technical Appendix). However, these findings should be interpreted with caution because, given the short duration of infectivity, even a low prevalence of *H. ducreyi* in a population with GUD implies that a reservoir of infected persons with a high rate of sex partners is present.

Recent research has identified *H. ducreyi* as a previously unrecognized cause of nongenital skin ulcers in tropical areas. In 2013–2015, six studies in Papua New Guinea (*3*,*6*), the Solomon Islands (*4*), Vanuatu (C.Y. Chen et al., pers. comm.), and Ghana (*5*; C.Y. Chen et al., pers. comm.) showed that a high proportion of laboratory-confirmed skin ulcers were caused by *H. ducreyi*. Nearly half of the 690 enrolled patients with ulcers in these 6 studies had *H. ducreyi* detectable by PCR, whereas other bacteria, such as *T. pallidum* subsp. *pertenue*, the causative agent of yaws, were detected in 25% of patients.

These cases of infection with *H. ducreyi* confirmed by molecular analysis suggest that clinicians should be more aware of this newly recognized bacterium in skin ulcers of persons in tropical areas. In the context of new efforts to eradicate yaws, mass treatment with azithromycin in Papua New Guinea reduced the absolute prevalence of ulcers not caused by yaws, which were mainly caused by *H. ducreyi*, from 2.7% to 0.6% (prevalence ratio 0.23, 95% CI 0.18–0.29) at 12 months after treatment (*6*). However, persistence of *H. ducreyi* at low levels after mass treatment in Papua New Guinea (*3*) and Ghana (*5*) suggest that 1 round of mass treatment might not be successful in eradicating *H. ducreyi* skin ulcers.

Our review has several limitations. First, the increase in HSV-related GUD as a result of immunosuppression by HIV infection would result in a decrease in the proportion of chancroid among all GUD case-patients. Second, the lack of sequential studies performed in similar clinical settings at multiple time points precludes an optimal interpretation of the apparent decrease. Third, results might be affected by poor-quality data from many developing countries and might be inflated by publication bias. Fourth, PCR is more sensitive than culture. Therefore, increasing diagnostic yield might have partially masked the scale of the decrease in *H. ducreyi* as a cause of GUD.

In summary, we observed a quantitative and sustained reduction in cases of chancroid as a result of antimicrobial drug syndromic management and major social changes. In addition, data from several research groups indicate that *H. ducreyi* can cause nongenital skin lesions in persons residing in different regions. Further studies of this newly described pathogen skin disease association are required, and appropriate policies are needed that include the routine practice of managing tropical skin ulcers.

**Technical Appendix.** Additional references on epidemiology of *Haemophilus ducreyi* infections.
