# Correction: Vol. 22, No. 1

**DOI:** 10.3201/eid2202.C12202

**Published:** 2016-02

**Keywords:** errata, erratum, correction

Some references were cited incorrectly in the print and initial PDF editions of Epidemiology of *Haemophilus ducreyi* Infections (C. González-Beiras al.). The online edition of the article is correct, and the online PDF has been corrected (http://wwwnc.cdc.gov/EID/article/22/1/14-0425_article).

