# Supplementary material for: Epidemiology of Haemophilus ducreyi Infections
Source: Emerg Infect Dis. 2016 Jan;22(1):1–8. doi: 10.3201/eid2201.150425 (PMC4696685; doi:10.3201/eid2201.150425)
Supplement: Supplementary file 1 — Technical Appendix. Additional references on epidemiology of Haemophilus ducreyi infections. [file 15-0425-Techapp-s1.pdf]

Article DOI: http://dx.doi.org/10.3201/eid2201.150425

Epidemiology of *Haemophilus ducreyi* Infections

Technical Appendix

References

41. Mertz KJ, Trees D, Levine W. Etiology of genital ulcers and prevalence of human immunodeficiency virus coinfection in 10 US cities. J Infect Dis. 1998;178:1795–8 PubMed. http://dx.doi.org/10.1086/314502

42. Sanchez J, Volquez C, Totten PA, Campos PE, Ryan C, Catlin M, et al. The etiology and management of genital ulcers in the Dominican Republic and Peru. Sex Transm Dis. 2002;29:559–67 PubMed. http://dx.doi.org/10.1097/00007435-200210000-00001

43. Behets FM, Brathwaite R, Hylton-Kong T. Genital ulcers: etiology, clinical diagnosis, and associated human immunodeficiency virus infection in Kingston, Jamaica. Clin Infect Dis. 1999;28:1086–90 PubMed. http://dx.doi.org/10.1086/514751

44. Bauwens JE, Orlander E, Gomez H. Epidemic Lymphogranuloma venereum during epidemics of crack cocaine use and HIV infection in the Bahamas. Sex Transm Dis. 2002;29:253–9 PubMed. http://dx.doi.org/10.1097/00007435-200205000-00001

45. Madani TA. Sexually transmitted infections in Saudi Arabia. BMC Infect Dis. 2006;6:3 PubMed. http://dx.doi.org/10.1186/1471-2334-6-3

46. Kyriakis KP, Hadjivassiliou M, Paparizos VA, Flemetakis A, Stavrianeas N, Katsambas A. Incidence determinants of gonorrhea, chlamydial genital infection, syphilis and chancroid in attendees at a sexually transmitted disease clinic in Athens, Greece. Int J Dermatol. 2003;42:876–81 PubMed. http://dx.doi.org/10.1046/j.1365-4362.2003.01737.x

47. Bruisten SM, Cairo I, Fennema H, Pijl A, Buimer M, Peerbooms PG, et al. Diagnosing genital ulcer disease in a clinic for sexually transmitted diseases in Amsterdam, The Netherlands. J Clin Microbiol. 2001;39:601–5. PubMed http://dx.doi.org/10.1128/JCM.39.2.601-605.2001

48. Mehta SD, Green SJ, Maclean I, Hu H, Bailey RC, Gillevet PM, et al. Microbial diversity of genital ulcer disease in men enrolled in a randomized trial of male circumcision in Kisumu, Kenya. PLoS ONE. 2012;7:e38991. PubMed http://dx.doi.org/10.1371/journal.pone.0038991

49. Phiri S, Zadrozny S, Weiss HA, Martinson F, Nyirenda N, Chen CY, et al. Etiology of genital ulcer disease and association with HIV infection in Malawi. Sex Transm Dis. 2013;40:923–8. PubMed http://dx.doi.org/10.1097/OLQ.0000000000000051

50. Zimba TF, Apalata T, Sturm WA, Moodley P. Aetiology of sexually transmitted infections in Maputo, Mozambique. J Infect Dev Ctries. 2011;5:41–7. PubMed

51. Tobias S, Shonhiwa S, Norbert F, Oke O, Ndjavera C, Chikukwa S, et al. Microbiological surveillance for sexually transmitted infections in Windhoek and Osahakati, Namibia. Sex Transm Infect. 2011;87:A103. http://dx.doi.org/10.1136/sextrans-2011-050108.10

52. O'Farrell N, Morison L, Moodley P, Pillay K, Vanmali T, Quigley M, et al. Genital ulcers and concomitant complaints in men attending a sexually transmitted infections clinic: implications for sexually transmitted infections management. Sex Transm Dis. 2008;35:545–9. PubMed http://dx.doi.org/10.1097/OLQ.0b013e31816a4f2e

53. Lewis DA, Müller E, Steele L, Sternberg M, Radebe F, Lyall M, et al. Prevalence and associations of genital ulcer and urethral pathogens in men presenting with genital ulcer syndrome to primary health care clinics in South Africa. Sex Transm Dis. 2012;39:880–5. PubMed http://dx.doi.org/10.1097/OLQ.0b013e318269cf90

54. Nilsen A, Kasubi MJ, Mohn SC, Mwakagile D, Langeland N, Haarr L. Herpes simplex virus infection and genital ulcer disease among patients with sexually transmitted infections in Dar es Salaam, Tanzania. Acta Derm Venereol. 2007;87:355–9. PubMed http://dx.doi.org/10.2340/00015555-0241

55. Suntoke TR, Hardick A, Tobian AA, Mpoza B, Laeyendecker O, Serwadda D, et al. Evaluation of multiplex real-time PCR for detection of *Haemophilus ducreyi, Treponema pallidum, herpes simplex virus* type 1 and 2 in the diagnosis of genital ulcer disease in the Rakai District, Uganda. Sex Transm Infect. 2009;85:97–101. PubMed http://dx.doi.org/10.1136/sti.2008.034207

56. Makasa M, Buve A, Sandøy IF. Etiologic pattern of genital ulcers in Lusaka, Zambia: has chancroid been eliminated? Sex Transm Dis. 2012;39:787–91. PubMed http://dx.doi.org/10.1097/OLQ.0b013e31826ae97d

57. Gomes Naveca F, Sabidó M, Amaral Pires de Almeida T, Araújo Veras E, Contreras Mejía Mdel C, Galban E, et al. Etiology of genital ulcer disease in a sexually transmitted infection reference center in Manaus, Brazilian Amazon. PLoS ONE. 2013;8:e63953. PubMed http://dx.doi.org/10.1371/journal.pone.0063953

58. Maan MA, Hussain F, Iqbal J, Akhtar S. Sexually transmitted infections in Pakistan. Ann Saudi Med. 2011;31:263–9. PubMed http://dx.doi.org/10.4103/0256-4947.81541

59. Hope-Rapp E, Anyfantakis V, Fouéré S, et al. Etiology of genital ulcer disease. A prospective study of 278 cases seen in an STD clinic in Paris. Sex Transm Dis. 2010;37:153–8. PubMed http://dx.doi.org/10.1097/OLQ.0b013e3181bf5a98

60. Mackay IM, Harnett G, Jeoffreys N, Bastian I, Sriprakash KS, Siebert D, et al. Detection and discrimination of herpes simplex viruses, *Haemophilus ducreyi*, *Treponema pallidum*, and *Calymmatobacterium* (*Klebsiella*) *granulomatis* from genital ulcers. Clin Infect Dis. 2006;42:1431–8. PubMed http://dx.doi.org/10.1086/503424

61. World Health Organization. Guidelines for the management of sexually transmitted infections. Geneva: The Organization; 2001.

62. Spinola SM, Ballard RC. Chancroid. In: Morse SA, Holmes KK, Ballard RC, editors. Atlas of sexually transmitted diseases and AIDS. 4th ed. Philadelphia: W.B. Saunders; 2010. p. 141–56.

63. Steen R, Elvira W, Kamali A, Ndowa F. Control of sexually transmitted infections and prevention of HIV transmission: mending a fractured paradigm. Bull World Health Organ. 2009;87:858–65. PubMed http://dx.doi.org/10.2471/BLT.08.059212
